# Properties of Boolean networks and methods for their tests

**DOI:** 10.1186/1687-4153-2013-1

**Published:** 2013-01-11

**Authors:** Johannes Georg Klotz, Ronny Feuer, Oliver Sawodny, Martin Bossert, Michael Ederer, Steffen Schober

**Affiliations:** 1 Institute of Communications Engineering, Ulm University, Albert-Einstein-Allee 43, 89081 Ulm, Germany; 2 Institute for System Dynamics, University of Stuttgart, 70569 Stuttgart, Germany

**Keywords:** Regulatory Boolean networks, Boolean networks, Linear threshold functions, Unate functions, Canalizing function, Sensitivity, Average sensitivity, Restricted functions, *Escherichia coli*

## Abstract

Transcriptional regulation networks are often modeled as Boolean networks. We discuss certain properties of Boolean functions (BFs), which are considered as important in such networks, namely, membership to the classes of unate or canalizing functions. Of further interest is the average sensitivity (AS) of functions. In this article, we discuss several algorithms to test the properties of interest. To test canalizing properties of functions, we apply spectral techniques, which can also be used to characterize the AS of functions as well as the influences of variables in unate BFs. Further, we provide and review upper and lower bounds on the AS of unate BFs based on the spectral representation. Finally, we apply these methods to a transcriptional regulation network of *Escherichia coli*, which controls central parts of the *E. coli* metabolism. We find that all functions are unate. Also the analysis of the AS of the network reveals an exceptional robustness against transient fluctuations of the binary variables.^a^

## Introduction

Boolean modeling is often used to describe signal transduction and regulatory networks [1-3]. Over the last years random Boolean models received much attention to find some generic properties that characterize regulatory networks. In addition to the study of topological features (e.g., [4]), the choice of Boolean functions in such networks is an important question to consider. Many results indicate the importance of functions with a low average sensitivity. For example, it is well known that a low expected average sensitivity is a prerequisite for non-chaotic behavior of random Boolean networks, e.g., [5,6]. Further, so called *canalizing* functions have been conjectured to be characteristic for biological networks [7]. These functions have a stabilizing effect on the network dynamics [1] and many functions occurring in (non-random) regulative networks are canalizing [7].

In this work we follow a non-random approach to find properties characterizing regulatory networks. Namely, we focus on the properties of Boolean functions in a large scale Boolean regulatory network model. Our goal is also to provide efficient algorithms to test these properties.

First, we consider the membership of the regulatory functions to certain classes of functions. We first consider unate functions, which are monotone in each of their variables and were shown to be implied by a biochemical model [2].

Next, we present a test using Fourier analysis to test canalizing properties of functions. Canalizing functions are used in signal processing for certain classes of filters [8] and play an important role in random and regulatory Boolean networks, as already mentioned. Interestingly, it has been shown in [9] that a subclass of canalizing functions, namely the nested canalizing functions, is identical to the class of unate-cascade functions, a subclass of the unate functions. The test presented in this work is inspired by [10], where the so-called forcing transform was introduced to test the membership of a function to the class of canalizing functions. Here, we generalize this approach to the Fourier transform, which is a more intuitive and natural approach and furthermore some spectral properties of canalizing functions have already been investigated in [11].

It is well known that the average sensitivity can be directly obtained from the Fourier spectral coefficients. Further, the Fourier transform turns out to be useful to prove bounds on the average sensitivity. We derive an upper bound for unate functions similar to known results for monotone functions and recall a well-known lower bound on the average sensitivity.

Finally, we apply our tests to a large-scale Boolean model of the transcriptional network of *Escherichia coli*. We extended the network model of the transcriptional network of *E. coli* (Covert et al. [3]) by mapping genes to their corresponding fluxes in the flux-balance model presented by [12]. The network has a layered feed-forward structure and shows characteristic topological features, such as a long-tail like out-degree distribution.

Throughout this article we use Fourier analysis to investigate the mentioned properties. In particular we use the concept of restricted functions. Therefore we derived both-way relations between the Fourier coefficients of a Boolean function and its restriction. A very general one-way approach of this relation can be found in [13].

The remainder of this article is organized as follows: In Section 2. we give a short introduction to Boolean functions and networks, discuss some fundamentals of Fourier analysis and investigate the spectra of restricted functions. In Section 3. we discuss certain classes and properties of Boolean functions and show efficient ways to check these properties. We also introduce the average sensitivity and prove an upper bound on it for unate functions. In Section 4. we finally introduce Boolean networks and apply our methods and tests to the regulatory network of *E.coli*. Some final remarks are given in Section 5.

## BFs

A BF *f*:{−1,1}^*n*^→{−1,1} maps *n*-ary binary *input* tuples to a binary *output*. In general, not all variables of a function *f* are *relevant*. A variable *i* is called relevant, if there exits at least one argument **x**∈{−1,1}^*n*^ such that *f*(**x**)≠*f*(**x**⊕*e*_*i*_), where the argument **x**⊕*e*_*i*_ is obtained from **x**by changing its *i*-th entry. In the following, we denote the number of relevant variables by *k*.

For the sake of simplicity we assume throughout this article, that *k*=*n*, i.e., all variables are relevant, but note that the expositions in Section 2.1 are valid in general. The assignment of + 1 and −1 chosen to represent the binary in and outputs is somewhat arbitrary. One can interpret the value −1 as “ON” or “TRUE” and + 1 as “OFF” or “FALSE”.

### Fourier analysis

Here we will give a short introduction to the concepts of Fourier analysis so far used in this article. Let us consider **x**=(*x*_1_,*x*_2_,…,*x*_*N*_) as an instance of a product distributed random vector **X**=(*X*_1_,*X*_2_,…,*X*_*N*_) with probability density function

$$P_{X}(x)=\underset{i}{\prod }P_{X_{i}}(x_{i}).$$

Furthermore, let *μ*_*i*_ be the expected value of *X*_*i*_, i.e., $\mu_{i}=E[X_{i}]$ and let $\sigma_{i}=\sqrt{1-\mu_{i}^{2}}$ be the standard deviation of *X*_*i*_. It can easily be seen that

(1) $$P_{X_{i}}(a_{i})=\frac{1+a_{i}\cdot \mu_{i}}{2}.$$

It is well known that any BF *f* can be expressed by the following sum, called Fourier-expansion [14,15],

(2) $$f(x)=\underset{U\subseteq [n]}{\sum }\hat{f}(U)\cdot \Phi_{U}(x),$$

where *n*={1,2,…,*n*} and

(3) $$\Phi_{U}(x)=\underset{i\in U}{\prod }\frac{x_{i}-\mu_{i}}{\sigma_{i}}.$$

For **U**=*∅* we define *Φ*_*∅*_(**x**)=1. The *Fourier coefficients*$\hat{f}(U)$ can be recovered by

(4) $$\hat{f}(U)=\underset{x}{\sum }P_{X}(x)\cdot f(x)\cdot \Phi_{U}(x).$$

Further, let **A**⊂**U**and $\bar{A}=U\setminus A$, then

(5) $$\Phi_{U}(x)=\Phi_{A}(x)\cdot \Phi_{\bar{A}}(x),$$

which directly follows from the definition of *Φ*_**U**_(Equation 3).

If the input variables *X*_*i*_ are uniformly distributed, i.e., *μ*_*i*_=0 and *σ*_*i*_=1, Equation (3) reduces to

$$\Phi_{U}(x)=\underset{i\in U}{\prod }x_{i},$$

and consequently, as *P*_**X**_(**x**)=2^−*n*^ for all **x**, Equation (4), reduces to

$$\hat{f}(U)=2^{-n}\underset{x}{\sum }f(x)\cdot \underset{i\in U}{\prod }x_{i}.$$

### Restricted functions

A function is called restricted, if some of the input variables are set to constants, i.e., variables *i*∈**K** are set to a constant *x*_*i*_=*a*_*i*_. Hence, the number of relevant variables is reduced by |**K**| . First, we consider the case that only one variable is restricted (**K**={*i*}). The function obtained in this way is denoted as

(6) $$f{|}_{x_{i}=a_{i}}:{\left\{-1,1\right\}}^{n-1}\to \{-1,1\}.$$

The following lemma gives a relation between the Fourier coefficients of the original function and its restriction.

**Proposition 1.** Let the function *f*(**x**) be a function in *n* variables. Consider the restricted function obtained by setting *x*_*i*_=*a*_*i*_, further, let ${\hat{f|}}_{x_{i}=a_{i}}$ be denoted as ${\hat{f}}_{a_{i}}$ then

(7) $${\hat{f}}_{a_{i}}(U)=\hat{f}(U)+\Phi_{\left\{i\right\}}(a_{i})\cdot \hat{f}(U\cup \{i\})$$

where **U**⊆[*n*]∖{*i*} and $\Phi_{\left\{i\right\}}(a_{i})=\frac{a_{i}-\mu_{i}}{\sigma_{i}}$.

*Proof.* Using Equation (4) we can rewrite (7) as

(8) $$\begin{array}{lll} {\hat{f}}_{a_{i}}(U)= & \underset{x}{\sum }P_{X}(x)f(x)\cdot \Phi_{U}(x)\; & \;\\ & +\Phi_{\left\{i\right\}}(a_{i})\cdot \underset{x}{\sum }P_{X}(x)f(x)\cdot \Phi_{U\cup \{i\}}(x).\; \end{array}$$

By applying (5) and (3) we get

$$\begin{array}{lll} \Phi_{U\cup \{i\}}(x) & =\Phi_{U}(x)\cdot \Phi_{\left\{i\right\}}(x)=\Phi_{U}(x)\cdot \frac{x_{i}-\mu_{i}}{\sigma_{i}}.\; & \; \end{array}$$

Hence, we can combine the two sums in (8) and obtain:

(9) $$\begin{array}{ll} {\hat{f}}_{a_{i}}(U) & =\underset{x}{\sum }\left(P_{X}(x)f(x)\cdot \Phi_{U}(x)\cdot \Xi_{i}\right),\; \end{array}$$

where

$$\Xi_{i}=\left(1+\frac{x_{i}-\mu_{i}}{a_{i}+\mu_{i}}\right)=\frac{a_{i}+x_{i}}{a_{i}+\mu_{i}},$$

due to $\Phi_{\left\{i\right\}}(a_{i})=\frac{a_{i}-\mu_{i}}{\sigma_{i}}=\frac{\sigma_{i}}{a_{i}+\mu_{i}}$.

Further, with $a_{i}=\frac{1}{a_{i}}$ and Equation (1) we get

$$\Xi_{i}=\left\{\begin{array}{ll} \frac{2}{1+a_{i}\cdot \mu_{i}}=\frac{1}{P_{X_{i}}(a_{i})}\; & \text{, if}x_{i}=a_{i}\\ 0\; & \text{, if}x_{i}=-a_{i}\\ \; \end{array}\right).$$

Thus, the sum in Equation (9) can be simplified to

$$\begin{array}{lll} {\hat{f}}_{a_{i}}(U) & =\underset{x|x_{i}=a_{i}}{\sum }\frac{P_{X}(x)}{P_{X_{i}}(a_{i})}\cdot f(x)\cdot \Phi_{U}(x)\; & \; \end{array}$$

and finally

$$\begin{array}{lll} {\hat{f}}_{a_{i}}(U) & =\underset{x|x_{i}=a_{i}}{\sum }P_{X|x_{i}}(x|a_{i})\cdot f(x)\cdot \Phi_{U}(x),\; & \; \end{array}$$

which is the definition of the Fourier coefficients from Equation (4) and concludes the proof. □

A closely related property is given by the following proposition. Please note that this result for uniform distributed input variables can also be retrieved using ([13], Lemma 2.17).

**Proposition 2.** Let *i*∈[*n*] be fixed and denote $f{|}_{x_{i}=a}$ with *f*_*a*_. For any *n*-ary BF *f*,

$$\begin{array}{lll} \hat{f}(U) & =\frac{1+\mu_{i}}{2}\left({\left(\Phi_{\left\{i\right\}}(+1)\right)}^{\left|U\cap \{i\}\right|}{\hat{f}}_{+1}(U\setminus \{i\})\right)\; & \;\\ & \;\;+\frac{1-\mu_{i}}{2}\left({\left(\Phi_{\left\{i\right\}}(-1)\right)}^{\left|U\cap \{i\}\right|}{\hat{f}}_{-1}(U\setminus \{i\})\right).\; & \; \end{array}$$

*Proof.* Starting from the definition we obtain

$$\begin{array}{lll} \hat{f}(U) & =E\left[f(X)\Phi_{U}(X)\right]\; & \;\\ & =P_{X_{i}}(+1)E\left[f(X)\Phi_{U}(X)|X_{i}=+1\right)]\; & \;\\ & \;\;+P_{X_{i}}(-1)E\left[f(X)\Phi_{U}(X)|X_{i}=-1\right]\; & \;\\ & =P_{X_{i}}(+1)\Phi_{\left\{i\right\}}(+1)E\left[f_{+1}(X)\Phi_{U\setminus \{i\}}(X)\right]\; & \;\\ & \;\;+P_{X_{i}}(-1)\Phi_{\left\{i\right\}}(-1)E\left[f_{-1}(X)\Phi_{U\setminus \{i\}}(X)\right].\; & \; \end{array}$$

Note that for *a*= + 1 or *a*=−1

$$E\left[f_{a}(X)\Phi_{U\setminus \{i\}}(X)\right]={\hat{f}}_{a}(U\setminus \{i\})$$

by definition, hence, the proposition follows from Equation (1). □

For the general case, that a BF is restricted to more than one input, the following Corollary to Proposition 1 applies:

**Corollary 1.** Let *f*(**x**) be a BF and $\hat{f}(U)$ its Fourier coefficients. Furthermore, let **K** be a set containing the indices *i* of the input variables *x*_*i*_, which are fixed to certain values *a*_*i*_. The Fourier coefficients of the restricted function are then given as

$${\hat{f|}}_{K}(U)=\underset{T\subseteq K}{\sum }\left(\Phi_{T}(a)\cdot \hat{f}(U\cup T)\right),$$

where **U** contains the indices for the Fourier coefficients of the restricted functions, i.e., **U**⊆[*n*]∖**K** and **a** is a vector containing all *a*_*i*_,*i*∈**K**.

## Classes and properties of functions

In this section, we will present and discuss some classes of BFs, namely unate and canalizing functions. Further, we will discuss properties of functions characterizing their *robustness*, like for example the AS.

### Unate functions

A BF is unate if it is monotone (either increasing or decreasing) in each of its variables, a precise definition will be given below. The class of unate functions is a simple extension of the class of monotone functions defined as follows

**Definition 1. ***A BF f*:{−1,1}^*n*^→{−1,1} is called monotone, if for each *i*∈{1,…,*n*} it holds that *f*(*x*_1_,…,*x*_*i*_=−1,…,*x*_*n*_)≤*f*(*x*_1_,…,*x*_*i*_=1,…,*x*_*n*_).

Now unate functions can be defined as follows.

**Definition 2. ***A BF f* is unate, if there exists a vector **a**∈{−1,1}^*n*^such that the function *f*(*a*_1_·*x*_1_,…,*a*_*n*_·*x*_*n*_) is monotone.

The class of unate functions is closed with respect to restriction, since every restriction of a locally monotone function yields again in a locally monotone function.

To test whether a function is unate or not it is sufficient to use the definition, however, a necessary condition for a function to be unate is given by the following proposition:

**Proposition 3** (for example [16]). If *f* is a unate function, then for each relevant variable *i*

$$\hat{f}(\{i\})\neq 0.$$

### Canalizing functions

A BF is called canalizing, if there exists a canalizing variable *x*_*i*_ and a Boolean value *a*_*i*_∈{−1,1} such that the function

(10) $$f{|}_{x_{i}=a_{i}}(x)=b_{i},$$

for all *x*_1_,…*x*_*i*−1_,*x*_*i* + 1_…*x*_*n*_, where *b*_*i*_∈{0,1} is a constant. If the restricted function, which is obtained by setting *x*_*i*_=1−*a*_*i*_, is again canalizing and so on, the function is called nested canalizing.

The following propositions give a relation between the Fourier coefficients and the canalizing property.

**Proposition 4. ***A BF f* is canalizing in variable *i*, if for any constants *a*_*i*_,*b*_*i*_∈{−1,1} the Fourier coefficients $\hat{f}(U)$ fulfill the following condition.

(11) $$\hat{f}(\emptyset )+\Phi_{\left\{i\right\}}(a_{i})\cdot \hat{f}(\{i\})=b_{i},$$

where *μ*_*i*_ is the expected value of *x*_*i*_and *σ*_*i*_ the corresponding standard derivation.

*Proof.* Obviously, if a function is canalizing, $E[f{|}_{x_{i}=a_{i}}(x)]=b_{i}$ holds. Since the expected value of a BF can be expressed as $E[f(x)]=\hat{f}(\emptyset )$ we obtain

$$E[f{|}_{x_{i}=a_{i}}(x)]={\hat{f|}}_{x_{i}=a_{i}}(\emptyset ).$$

Using Proposition 1, we get

$$E[f{|}_{x_{i}=a_{i}}(x)]=\hat{f}(\emptyset )+\Phi_{\left\{i\right\}}(a_{i})\cdot \hat{f}(\{i\}),$$

and the proposition follows from Equation (11). □

A similar result namely the calculation of the Fourier coefficients of a canalizing BF from the coefficients of the restricted functions $\hat{f}{|}_{x_{i}=a_{i}}(U)$ is addressed in [11]. These results can also be achieved using Proposition 2.

Proposition 4 can easily be extended for nested canalizing functions:

**Proposition 5. ***Assume f*(*x*) is canalizing for variables *x*_*i*_=−*a*_*i*_,*i*∈**K**, then *f*(*x*) is canalizing for *x*_*j*_=*a*_*j*_,*j*∉**K**, i.e., $E[f_{K\cup \{j\}}(x)]=b_{j}$, if

$$\underset{T\subseteq K\cup \{j\}}{\sum }\left(\underset{i\in T}{\prod }\left(\Phi_{\left\{i\right\}}(a_{i})\right)\cdot \hat{f}(T)\right)=b_{j}.$$

*Proof.* The proof follows from Corollary 1 and Proposition 4. □

From Proposition 4 it is clear that the canalizing property can be tested by considering all Fourier coefficients of order one. Using the Fast Walsh Transform [17] this test is as fast as the one presented in [10], however, once we have retrieved the spectra of a function, we can easily compute other properties, such as the AS (see next section).

### AS of functions

The AS [18] gives the influence of random disturbance at the input on the output of a BF. This can be interpreted as an indicator for the robustness of this BF and finally for the whole Boolean network.

To define the as we first have to look at the sensitivity *s*_**x**_(*f*) of an input argument **x**∈{0,1}^*n*^. It is defined as the number of single bit-flips in **x** so that the output of the function will change, i.e., *s*_**x**_(*f*) is number of variables *i* for which *f*(**x**)≠*f*(**x**⊕*e*_*i*_). The AS *as*(*f*) is the expected value over all arguments **x**:

(12) $$\text{as}(f)=E_{x}[s_{x}(f)].$$

It is worth noting that the as depends on the distribution of the input vector. For example, a function having a low AS for the uniform distribution may have a large AS for other distributions. In general, the AS can be as large as the number of relevant variables *k*, i.e.,

0≤as(f)≤k.

**Figure 1:**
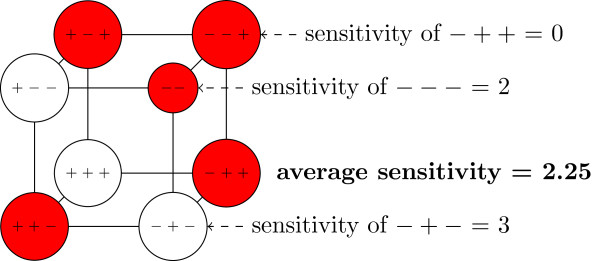
**Sensitivities and AS of an exemplary BF.** Each node represents an argument of a BF with *n*=3 variables, where + stands for + 1 and − represents a −1. A blank node indicates that the corresponding output of the function is 1 while a shaded node represents a −1. The sensitivity of a node is then the number of neighbor-nodes with a different shading. The expected value of these sensitivities is the AS.

Figure 1 explains the concepts defined above at an example.

Alternatively, the AS can be defined using the notion of *influence*. The influence *I*_*i*_(*f*) of a single input variable *i* on the functions *f* is defined as

(13) $$I_{i}(f)=\text{Prob}[f(X)\neq f(X⊕e_{i})].$$

The AS can then be defined as the sum of all influences [19]

(14) $$\text{as}(f)=E_{x}[s_{x}(f)]=\underset{i}{\sum }I_{i}(f).$$

The influence *I*_*i*_(*f*) for a unate function *f* is directly related to the corresponding Fourier coefficient:

(15) $$I_{i}(f)=\frac{\left|\hat{f}(\{i\})\right|}{\sigma_{i}},$$

as it was shown for monotone functions in ([16], Lemma 4.5) and can easily be extended to unate functions. Note that Equation (15) directly gives a proof for Proposition 3. Hence, for unate functions we can write

(16) $$\text{as}(f)=\overset{n}{\underset{i=1}{\sum }}\frac{\left|\hat{f}(\{i\})\right|}{\sigma_{i}}=\sqrt{{\left(\overset{n}{\underset{i=1}{\sum }}\frac{\left|\hat{f}(\{i\})\right|}{\sigma_{i}}\right)}^{2}},$$

and from the Cauchy-Schwarz inequality it follows that

(17) $$\begin{array}{lll} \text{as}(f) & \leq \sqrt{\overset{n}{\underset{i=1}{\sum }}{\left(\left|\hat{f}(\{i\})\right|\right)}^{2}}\sqrt{\overset{n}{\underset{i=1}{\sum }}{\left(\frac{1}{\sigma_{i}}\right)}^{2}}\; & \;\\ & \leq \sqrt{\left(1-\hat{f}{\left(\emptyset \right)}^{2}\right)}\sqrt{\overset{n}{\underset{i=1}{\sum }}{\left(\frac{1}{\sigma_{i}}\right)}^{2}}.\; \end{array}$$

Together with a lower bound as presented in [19,20] and since $1-\hat{f}{\left(\emptyset \right)}^{2}=1-E{\left[f\right]}^{2}=\text{Var}(f)$ we obtain the following proposition.

**Proposition 6. ***Let f be an unate BF with in-degree n, further let σ_i_ be the standard derivation of the i-th input, then the AS of f(as(f)) is bounded by*

(18) $$\begin{array}{ll} \text{Var}(f)\leq \text{as}(f)\leq \sqrt{\text{Var}(f)}\sqrt{\overset{n}{\underset{i=1}{\sum }}{\left(\frac{1}{\sigma_{i}}\right)}^{2}}, & \; \end{array}$$

where Var(f) denotes the variance off.

It can be shown that some functions get close to the upper bound. Assuming uniform distribution the upper bound in Equation (18) is smaller than $\sqrt{n}$. But it is well known that the AS of the majority function behaves like $O(\sqrt{n})$ (see for example [21]).

## Application to a regulatory network of *E.coli*

In the previous sections, we only considered BFs. Now we will focus on BNs. A synchronous BN of *N* nodes can be described by a graph *G*=*G*(*V*,*E*) with nodes *V*⊆[*N*], |*V*|=*N*, and edges *E*⊆*V*×*V*, and a set of ordered BFs *F*=(*f*_1_,*f*_2_,…,*f*_*N*_), where we also allow a *dummy* function (see below). Each *f*_*i*_has *n*_*i*_=*k*_*i*_=in-deg(i) relevant variables where in-deg(i) is the in-degree of node *i*, i.e., the number of edges (*j*,*i*) with *j*∈*V*. In this case a node *j* is called a *controlling* node of *i*. If a node *i* has in-degree zero, the dummy function is attached and we call it an in-node. Consequently, the number of edges emerging from *i* is called the out-degree of node *i*. Usually to each node a binary state variable is assigned, i.e., for node *i* we assign *x*_*i*_(*t*)∈{−1, + 1}. For in-nodes the state can be set by some external process at some time *t*_0_. The state of all other nodes at time *t* depend on its BF and the states of all controlling nodes at time instant *t*−1.

**Figure 2:**
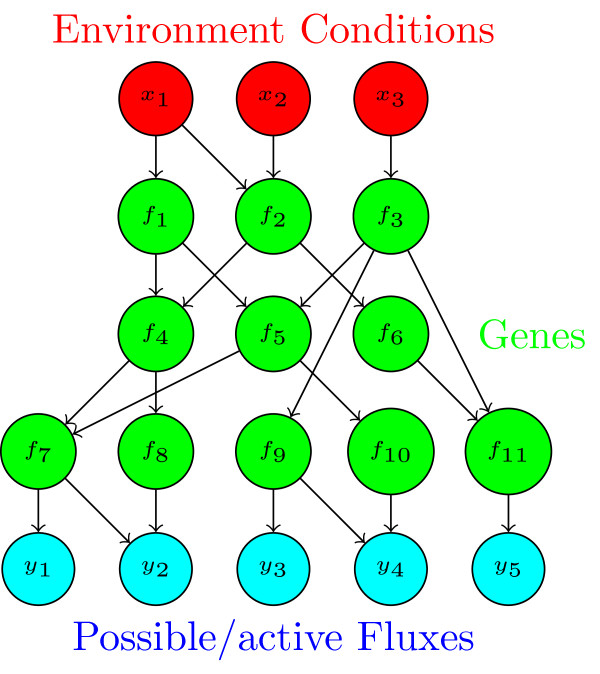
**Example of a layered feed-forward Boolean network.** The picture shows an example network. The upper layer (in red) consists of the inputs. These are fed forward through the middle layers (representing the regulation of the genes, in green) to the lowest layer. This layer is the output of the network (in blue). In our case it represents the fluxes of the metabolism.

In this article, we are only considering feed-forward networks, i.e., networks without *feedback loops*. In such feed-forward BNs, the set of nodes is partitioned in layers *L*_1_,*L*_2_,…,*L*_*l*_. If a node *i* is an element of layer *L*_*h*_ all controlling nodes are element of layers *L*_*m*_ with *m*<*h*. The first (highest) layer *L*_1_ consists of the input nodes (in-nodes), while the lowest layer *L*_*l*_consists of the output nodes (out-nodes). In Figure 2 a sample network is depicted.

### Structural properties

We applied the tests described in the previous sections to the regulatory network of *E. coli*[3]. The model provides Boolean formulas that describe how environmental conditions act on gene expression via a transcriptional regulatory network. We extended this network by the mapping of the genes to their corresponding fluxes in the flux-balance model [12]. The network as described in the literature contains functions with irrelevant variables, respectively, redundant edges, which are removed. A list of the affected nodes and the removed edges can be found in the Additional file 1.

**Figure 3:**
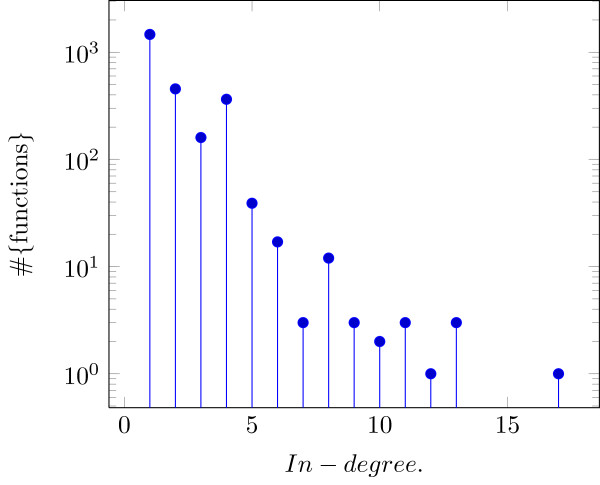
**In-degree distribution of the investigated network ([**[3]**] extended by [**[12]**]).**

**Figure 4:**
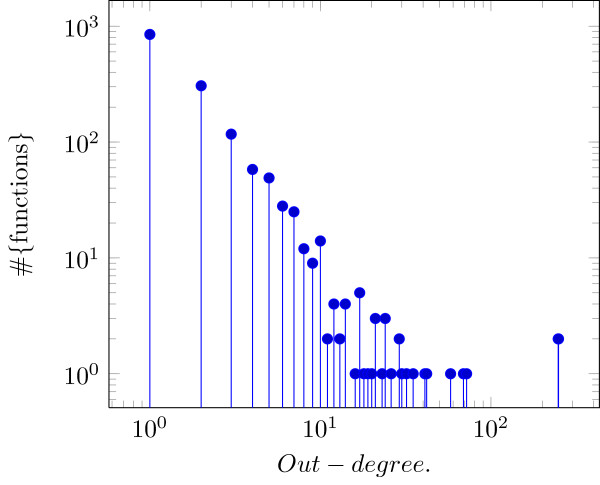
**Out-degree distribution of the investigated network ([**[3]**] extended by [**[12]**]).**

The resulting network has a total of *N*=3915 nodes and |*E*|=4874 edges, where 1,386 of these nodes are in layer *L*_1_, i.e., are inputs, hence, 2,529 nodes have a non-dummy function attached. The in-degree and out-degree distributions can be found in Figures 3 and 4. The average in-degree is 1.92724. The out-degree distribution shows a typical long tail behavior [4].

We found that all functions attached to the nodes are unate. Furthermore 2499 functions (98.8%) are canalizing An overview of the functions, which are not canalizing, can be found in the Additional file 1.

### Robustness

**Figure 5:**
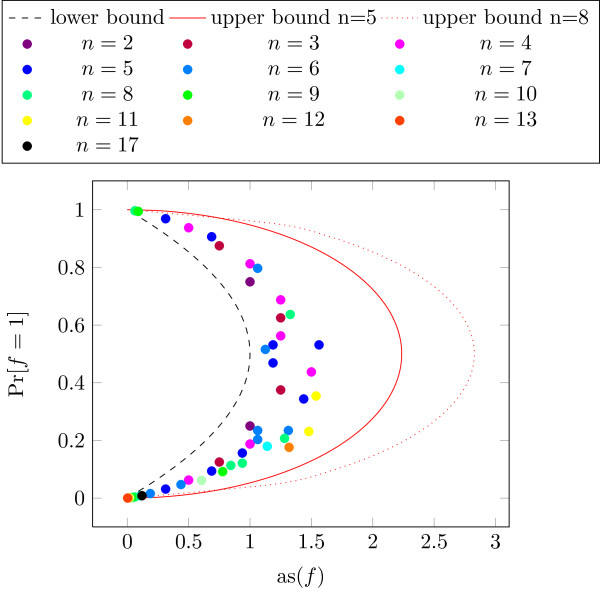
AS of functions plotted versus bias of functions (equally distributed inputs).

To evaluate the robustness of the network we assume in general that the state of nodes can be described by binary random variables. In a first step we assumed that each random variable of each nodes is uniformly distributed. This implies that we consider each node independently, i.e., the topology of the network is ignored. We calculated the AS for all functions in the network. In Figure 5, the resulting AS is plotted versus the *bias*, which is the probability that the output of the function equals one (a similar analysis appears in [22]). Each color represents a BF with a certain in-degree *n*. We also included the lower bond and two exemplary upper bounds for *n*=5 and *n*=8 (Equation (18)). For increasing *n* the upper bounds will grow, i.e., the bound will move further to the right.

Obviously, functions with a strong bias, i.e., with a high probability to be either −1 or 1, have a low AS. Further it can be seen that the average sensitivities of all functions are very close to the lower bound. The mean value of the AS is 0.918874. Hence, it can be stated that the AS of this network is rather low. Similar results can be obtained considering the network without the extension as originally defined by Covert et al. [3] and Samal and Jain [23].

In a second step we want to take the topology of the network into account. Therefore, we now assume that only the in-nodes of the network are equally distributed. However, the output of these functions will most certainly not be uniform, i.e., the functions have a bias unequal zero. Since the outputs of these functions serve as inputs of the functions of the next layer, we assume that their input distributions follow the output distribution of the first-layer functions. The output distributions of the second-layer functions serve then as input distributions of the third layer and so on. Obviously this has an impact on the *as* of the functions.

**Figure 6:**
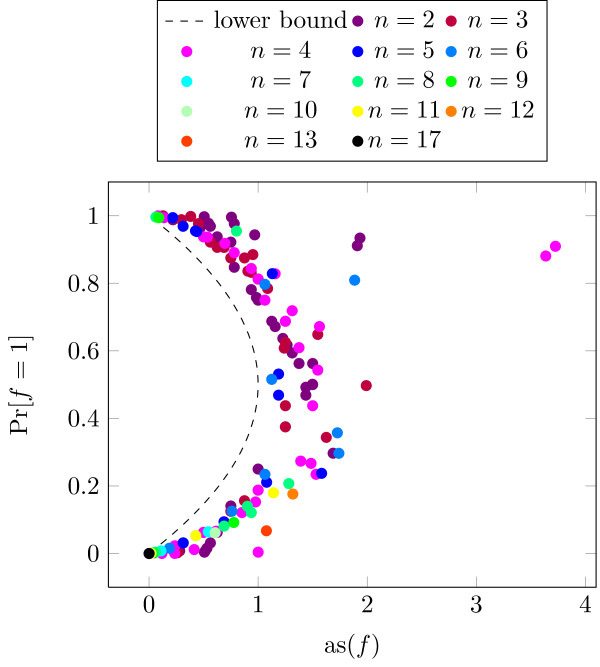
AS of functions plotted versus bias of functions (product distributed inputs).

The results are shown in Figure 6. We did not include any upper bounds in this figure since these now depend on each input distribution (see Proposition 6). It can be seen that the AS is still very close to the lower bound. However, a few functions have a rather large AS, e.g., it can be seen in Figure 6 that two types of functions with in-degree *K*=2 are very close to their upper bound (which is in this case at *as*(*f*)=2). These functions have an argument with a sensitivity of 2. Due to the input distribution of these functions this argument has a very large probability (>98*%*) which leads to a very high AS close to 2. Such high AS are normally observed for XOR and related functions. The average value of the AS is 0.908445, hence the AS of the network further decreases when applying product distributions at the inputs of the functions.

### Comparison with random ensembles

The network appears to be more *robust* against transient errors as for example certain randomly constructed networks. The in-degree distributions of all controlled nodes (in-degree larger zero) is shown in Figure 3. For all nodes with in-degree *k* we choose a random function out of the set of functions with *k* relevant variables. For *k*=1 this results in E[as(f)]=1, for *k*>1 we can at least state that $E[\text{as}(f)]>\frac{k}{2}$, as it is well known that if we choose randomly from functions, we expect an AS of $\frac{k}{2}$. Taking the in-degree distribution into account this implies that the expectation of the AS of all BFs chosen in this way is larger than 1.25.

It is well known that random function ensembles with lower expected AS can be constructed, if functions with a higher bias have higher probability to be chosen [24]. To test if the observed robustness can be explained due to the bias of the functions, we proceeded as follows. Again, a random function is chosen for each node with in-degree *k*. We determine the frequency distribution for the bias *b*=*Pf*=1] for all functions of the original network model with a certain in-degree *k*. The random network is generated by replacing the original functions of the network with functions drawn from an ensemble of functions with the same distribution. For example, if *k*=2, roughly 32% of all functions have *b*=0.25, while all others have *b*=0.75. Hence, with probability 32% we choose a function with *b*=0.25, and *b*=0.75 otherwise. The data can be found in the Additional file 1. As shown in [25,26], the expectation of the AS is then given by

(19) $$E(\text{as}(f))=2\text{kb}(1-b)\frac{2^{k}}{2^{k}-1}.$$

The results obtained are shown in Table 1 sorted according to the in-degree *k*. For *k*=1 and *k*=2 the observed mean of *as*(*f*) and the expectation of the random function coincides as only identity functions, respectively, AND or OR functions are chosen. For larger *k*, the observed mean is always smaller as the expectation of the random function. For some values of *k*, for example *k*=9, both values are close to each other. This is due to the fact that the corresponding functions are highly biased, which means that there are three existing functions with the values for *b* being 0.00195312, 0.0917969 and 0.994141. In contrast, for *k*=11 the mean of the observed values and the expectation are far from each other. Indeed out of three functions, there is one function with *b*=0.354004 for which, according to Equation (19), the expectation of the AS is 5.03353147.

**Table 1:** **Fraction of functions with in-degree*k*, the mean of the AS of all functions with in-degree*k*, and the expectation of an accordingly chosen random function with same in-degree and same bias distribution (see text and Equation**19**)**

***k***	**Fraction of functions**	**av(*f*)**	E(av(f)
1	0.579905	1.000000	1.000000
2	0.179984	1.000000	1.000000
3	0.063291	0.887500	0.985714
4	0.143987	0.572115	0.623077
5	0.015427	0.491987	0.659895
6	0.006725	0.933824	1.737920
7	0.001187	0.796872	1.423026
8	0.004747	0.760416	1.641421
9	0.001187	0.300781	0.547935
10	0.000791	0.312500	0.587713
11	0.001187	1.009441	2.984577
12	0.000396	1.318360	3.481815
13	0.001187	0.003174	0.003174

It should be noted that in random BNs the expectation of the AS is an *order parameter*[5,6]. That is, if the expectation is less or equal to one many random networks show the so-called ordered behavior. Namely, single transient errors introduced in network nodes (by flipping their state) do not spread through the network with high probability. This ordered behavior is in sharp contrast to the so-called disordered behavior of random networks which is characterized by an expectation of the AS larger one. Indeed, it has been conjectured that biological relevant networks should be ordered (or critical) but not disordered [27]. A further investigation on how canalizing and nested canalizing functions influence the average sensitivity can be found in [7,11].

### Impact of mutations on the metabolism

When investigating a regulatory network, the impact of the network on the metabolism is of major interest. Hence, only the stability of nodes in the bottom layer, i.e., the output of the network, is relevant. In regulatory networks, mutations are a source for errors. We consider two possible types of mutations. First we assume that a part of promoter region of a gene is mutated or deleted. In terms of our network this means that a edge is removed and the corresponding input is set to false (+ 1). The gene may still be transcribed, hence, the node itself remains functional. The second type of mutation is the deletion of a gene or a mutation which leads to disfunctional gene. In this case, the node is constantly set to false. In both cases, the value of one node may change (error). This error is now fed through the out-going edges of this node to other nodes. However, due to the low sensitivity of all functions in the network, the error has no impact on many nodes and, therefore, will in most cases not reach the bottom layer, which is, as mentioned above, the only part of the network, whose stability is crucial. From that point of view it can be stated that these permanent errors behave similar to the transient errors described above and that networks with a low mean AS are robust against such errors.

## Summary

It is an important problem to characterize BFs that appear in Boolean models of regulatory networks. This will help to understand the constraints underlying such networks, but can, for example, also help to improve network inference algorithms (see for examples [28,29] for algorithms that utilize the membership to the class of unate functions). In this study, we focused on several properties that have been shown to be of interest in the context of Boolean regulatory networks. Namely, we discussed different classes of BFs such as unate and canalizing functions. Further, sensitivity measures of BFs, like the influence of variables, or the AS are considered. We devised simple algorithms to test these properties. To test canalizing properties of BFs we applied the Fourier representation of BFs where functions are represented as multivariate, multilinear real polynomials. To this end, we introduced two spectral relationships between the so-called *restricted* BFs and their unrestricted counter part. The Fourier representation is further useful as many interesting properties such as the influence of unate functions or the AS of BFs can easily be characterized in the spectral domain. For example, we show how to obtain theoretical upper bounds on the AS for unate functions using spectral techniques.

As an application of our results, we analyzed an extended [30] regulatory Boolean network model of the central metabolism of *E. coli*. It turned out that most functions are within the classes of unate functions. Further, the AS of most functions is close to a theoretical lower bound and far from the new upper bound. Especially, functions with large in-degree have low AS even if their so-called bias is close to 0.5 (see Figure 5). We compared our findings to random BNs with similar parameters and find that the investigated networks has an even lower AS. From that we conclude that the whole network is stable, and robust to small changes, e.g., mutations.

## Endnote

^a^Preliminary results of this study have been presented at the 8th International Workshop on Computational Systems Biology (WCSB 2011) and the 3rd International Conference on Bioinformatics and Computational Biology (BICoB 2011).

## Competing interests

The authors declare that they have no competing interests.

## Supplementary Material

Additional file 1This.xls file contains 3 sheets with listings of:Click here for file

## References

[B1] Kauffman S Peterson C Samuelsson B Troein C Genetic networks with canalyzing Boolean rules are always stable Proc. Natl Acad. Sci. USA 2004 101 49 17102 17107 10.1073/pnas.0407783101 15572453 PMC534611

[B2] Grefenstette J Kim S Kauffman S An analysis of the class of gene regulatory functions implied by a biochemical model Biosystems 2006 84 2 81 90 http://www.sciencedirect.com/science/article/B6T2K-4HWXP4R-3/2/61a3092f98470e99a2c33786416697d0 10.1016/j.biosystems.2005.09.009 16384633

[B3] Covert MW Knight EM Reed JL Herrgard MJ Palsson BO Integrating high-throughput and computational data elucidates bacterial networks Nature 2004 429 6987 92 96 http://dx.doi.org/10.1038/nature02456 10.1038/nature02456 15129285

[B4] Aldana M Boolean dynamics of networks with scale-free topology Physica D 2003 185 45 66 10.1016/S0167-2789(03)00174-X

[B5] Shmulevich I Kauffman SA Activities and sensitivities in Boolean network models Phys. Rev. Lett 2004 93 4 048701 15323803 10.1103/PhysRevLett.93.048701 PMC1490311

[B6] Mahdavi byK, Culshaw R, Boucher J Dynamics of random Boolean networks 2007 World Scientific Publishing Co, Singapore

[B7] Harris SE Sawhill BK Wuensche A Kauffman S A model of transcriptional regulatory networks based on biases in the observed regulation rules Complexity 2002 7 4 23 40 10.1002/cplx.10022

[B8] Gabbouj M Yu PT Coyle EJ Convergence behavior and root signal sets of stack filters Circuits Syst. Signal Process 1992 11 171 193 10.1007/BF01189226

[B9] Jarrah AS Raposa B Laubenbacher R Nested Canalyzing, Unate Cascade, and Polynomial Functions Physica D 2007 233 2 167 174 10.1016/j.physd.2007.06.022 18437250 PMC2330334

[B10] Shmulevich I Lahdesmaki H Egiazarian K Spectral methods for testing membership in certain post classes and the class of forcing functions. Signal Process Lett. IEEE 2004 11 2 289 292

[B11] Kesseli J Rämö P Yli-Harja O Analyzing dynamics of Boolean networks with canalyzing functions using spectral methods Proceedings of the 2005 International TICSP Workshop on Spectral Methods and Multirate Signal Processing (SMMSP 2005) (Riga, Latvia, 20-22 June 2005) 151 158

[B12] Feist AM Henry CS Reed JL Krummenacker M Joyce AR Karp PD Broadbelt LJ Hatzimanikatis VBO Palsson V A genome-scale metabolic reconstruction for Escherichia coli K-12 MG1655 that accounts for 1260 ORFs and thermodynamic information Mol. Syst. Biol 2007 3 121 10.1038/msb4100155 17593909 10.1038/msb4100155 PMC1911197

[B13] Bernasconi A Mathematical techniques for the analysis of Boolean functions. PhD thesis 1998 University of Pisa, Italy

[B14] Bahadur RR Solomon byH A representation of the joint distribution of responses to n dichotomous items Studies on Item Analysis and Prediction, no. 6 in Stanford Mathematical Studies in the Social Sciences 1961 Stanford University Press, Stanford, CA 158 176

[B15] Furst ML Jackson JC Smith SW Improved learning of AC0 functions Proceedings of the Fourth Annual Workshop on Computational Learning Theory 1991 Morgan Kaufmann Publishers Inc., Santa Cruz 317 325

[B16] Bshouty NH Tamon C On the Fourier spectrum of monotone functions J. ACM 1996 43 4 747 770 10.1145/234533.234564

[B17] Shanks J Computation of the fast Walsh-Fourier transform IEEE Trans. Comput 1969 C-18 5 457 459

[B18] Benjamini I Kalai G Schramm O Noise sensitivity of Boolean functions and applications to percolation Publications mathematiques de l’IHES 1999 90 5 43

[B19] Kahn J Kalai G Linial N The influence of variables on Boolean functions Proceedings of the 29th Annual Symposium on Foundations of Computer Science White Plains, (New York, USA, 24-26 Oct 1988) 68 80

[B20] Friedgut E Boolean functions with low average sensitivity depend on few coordinates Combinatorica 1998 18 27 35 10.1007/PL00009809 10.1007/PL00009809

[B21] O’Donnell R Some topics in analysis of boolean functions Proceedings of the 40th annual ACM symposium on Theory of computing 2008 (ACM, Victoria 569 578 http://portal.acm.org/citation.cfm?id=1374458

[B22] Heckel R Schober S Bossert M Harmonic analysis of Boolean networks: determinative power and perturbations 2011 arXiv:1109.0807 10.1186/1687-4153-2013-6 PMC3748841 23642003

[B23] Samal A Jain S The regulatory network of E. coli metabolism as a Boolean dynamical system exhibits both homeostasis and flexibility of response BMC Syst. Biol 2008 2 21 http://dx.doi.org/10.1186/1752-0509-2-21 10.1186/1752-0509-2-21 18312613 PMC2322946

[B24] Derrida B Pomeau Y Random networks of automata—a simple annealed approximation Europhys. Lett 1986 2 45 49 10.1209/0295-5075/2/1/007

[B25] Schober S Analysis and identifiation of Boolean networks using harmonic analysis 2011 Dissertation, Ulm University, Ulm, Germany

[B26] Schober S Bossert M Analysis of random Boolean networks using the average sensitivity 2007 arXiv:nl.cg/0704.0197

[B27] Kauffman SA Metabolic stability and epigenesis in randomly constructed nets J. Theor. Biol 1969 22 437 467 10.1016/0022-5193(69)90015-0 5803332

[B28] Schober S Kracht D Heckel M Bossert R Detecting controlling nodes of Boolean regulatory networks EURASIP J. Bioinf. Syst. Biol 2011 27 11 1529 1536 http://www.ncbi.nlm.nih.gov/pubmed/21989141 10.1186/1687-4153-2011-6 PMC3377916 21989141

[B29] Maucher M Kracher B Kühl M Kestler HA Inferring Boolean network structure via correlation Bioinformatics 2011 http://bioinformatics.oxfordjournals.org/content/early/2011/04/05/bioinformatics.btr166.abstract 10.1093/bioinformatics/btr166 21471013

[B30] Feuer R Gottlieb K Klotz JG Schober S Bossert M Sawodny O Sprenger G Ederer M Model-based analysis of adaptive evolution Proceedings of the 8th International Workshop on Computational Systems Biology (WCSB) 2011 (Zuerich, Switzerland 108 111

